# To Be Oats or Not to Be? An Update on the Ongoing Debate on Oats for Patients With Celiac Disease

**DOI:** 10.3389/fped.2019.00384

**Published:** 2019-09-26

**Authors:** Inna Spector Cohen, Andrew S. Day, Ron Shaoul

**Affiliations:** ^1^Pediatric Gastroenterology and Nutrition Institute, Ruth Children's Hospital of Haifa, Rambam Medical Center, Faculty of Medicine, Technion, Haifa, Israel; ^2^Department of Paediatrics, University of Otago, Christchurch, New Zealand

**Keywords:** oats, celiac, safety, adherence, small bowel

## Abstract

To date, the only known effective treatment for celiac disease (CD) is a strict gluten-free diet (GFD) for life. Patients with CD often find it difficult to adhere to strict GFD. Oats, compared with wheat, barley, and rye, contain less amounts of prolamins. Inclusion of oats in a GFD might be valuable due to their nutritional and health benefits and increase of food variety. Therefore, they may potentially improve feeding diversity for these children and improve taste and satiety. We reviewed the literature to evaluate the safety of oats in CD patients. We have searched PUBMED, societal guidelines and national health authorities' recommendations. The following aspects were reviewed: gastrointestinal symptoms, malabsorption, serology including specific avenin antibodies, mucosal changes, avenin toxicity, immunogenicity of oats, and quality of life. We also referred to wheat contamination of oat products, the safe amount of oats for CD patients and the type of oats recommended. Data support that pure oats are well-tolerated by most CD patients, at moderate amounts (20–25 g/day dry rolled oats for children; 50–70 g/day for adults). Nevertheless, since the potential for sensitivity/toxicity exists, oats should be added with caution to a GFD, only after all CD symptoms including weight loss and growth disturbances have resolved, after at least 6 months of conventional GFD and probably also after normalization of serology. The need for pre exposure biopsy is unclear and should be considered on an individual basis.

## Introduction

Children with celiac disease (CD) often find it difficult to adhere to a strict gluten free diet (GFD) (1). Oats, compared with wheat, barley and rye, contain less amounts of prolamins (10 vs. 33–50%) (2), which are the gluten components responsible for the toxicity noted in susceptible individuals. Therefore, inclusion of oats in a GFD may potentially improve feeding diversity for these children (1), and also improve taste and satiety (3).

The GFD may be low in fiber, iron, folate, calcium, magnesium, zinc, and B-complex vitamins (thiamin, riboflavin, niacin, and vitamin B12), as well as vitamin D. Kilned and unkilned varieties of oats can improve vitamin B1, magnesium, and zinc intake in patients with CD in remission, and thus provide a significantly better nutritional profile compared to regular GFD (4). Oats also represent a good source of fiber in a GFD. Betaglucan in oats lowers postprandial plasma glucose and attenuates insulin responses. In addition, it increases bile acid excretion and transport with subsequent lowering of low-density lipoproteins. Oats contain about twice as much protein as rice. Oats also contain 6–8% of oil with a high proportion of unsaturated fatty acids. In addition, avenanthramides (specific antioxidants) are also present in oats (4).

Since Dicke's studies many decades ago (5, 6) there has been a controversy over the inclusion of oats in GFD. Early studies on patients with CD suggested intestinal malabsorption and exacerbation of abdominal symptoms after ingestion of oats (7, 8). More recent *in vitro* and *in vivo* studies have re-questioned the toxicity of oats and have suggested that inclusion of oats in a GFD may be reasonable and safe. This report considers the current evidence for oats in individuals with CD.

## Gastrointestinal Symptoms Following Ingestion of Oats

Baker and Read (8) measured urinary excretion of an oral 5 g xylose load before and 4 weeks after an oatflakes challenge in 11 adult patients and one child with CD on GFD for at least 6 months. They noted that six patients developed either gastrointestinal (GI) symptoms or anorexia and irritability; three of them had reduction in xylose excretion to between 47 and 77% of the pre-challenge levels.

Peräaho et al. (9) randomized 39 patients with CD on a GFD without oats who had documented mucosal recovery (though not complete in all cases) either to consume 30 g of oats-containing gluten-free products daily or to continue their regular diet without oats. The follow-up time was 1 year. They observed a trend toward a higher Gastrointestinal Symptom Rating Scale (GSRS) in the oats group, and the symptoms of diarrhea were more severe in the oats group at the end of the study (statistically significant). The constipation score increased similarly in both groups, while indigestion symptoms were improved in both groups, but more effectively in patients taking oats.

Koskinen et al. (10) enrolled 23 children with CD (ages 7–18 years) who maintained a conventional GFD (including abstinence of oats) for at least 2 years. Thirteen children were then randomized to undergo an open oats challenge and 10 had a gluten challenge, allowing the consumption of wheat, rye, and barley in addition to oats. Median daily oats consumption was 45 g/day. During the 2 years trial two children who ingested oats, but not gluten, developed dramatic GI symptoms but without signs of immune activation or CD relapse on small bowel biopsies.

Størsrud et al. (11) followed 20 adults with CD in remission who were taking a daily intake of 100 g of uncontaminated rolled oats for a period of 2 years. Flatulence was the most pronounced reported symptom achieving maximum intensity at 6 months.

Although these studies showed symptomatology in patients with CD exposed to oats, this is not the case in other studies. For example, Janatuinen et al. (12) followed 52 adult patients with CD in remission and 40 patients with newly diagnosed CD for a period of 12 months. They divided each group into two: those consuming oats and those who did not. The mean oat intake in the oat group was 45 g daily. They found that the use of oats by adult patients with CD in remission as part of a GFD had no unfavorable effects, and did not prevent symptomatic healing in patients with newly diagnosed disease.

Furthermore, Gatti et al. (13) in their 15 months double-blind, randomized, placebo-controlled multicenter study, enrolled 306 children with CD who had not previously consumed oats. They were randomized into two groups following either A-B treatment (6 months of diet “A,” 3 months of standard GFD, 6 months of diet “B”), or B-A treatment (6 months of diet “B,” 3 months of standard GFD, 6 months of diet “A”). A and B diets included gluten-free (GF) products with either purified oats or placebo, respectively. They monitored GI symptoms, growth data, and intestinal permeability tests (IPT) with measurement of urinary lactulose/mannitol (L/M) ratio. They found that the prolonged intake of a considerable amount of daily oats did not cause any change in clinical symptoms or intestinal permeability. Nevertheless, the specific amount of oats ingested was not documented in this study.

Sey et al. (14) challenged 15 adults with CD with 350 g/week of pure, uncontaminated oats for 12 weeks. The patients had been asymptomatic on a GFD for at least 1 year and had a normal tissue transglutaminase (tTG) level at the entry to the trial. They were largely asymptomatic throughout the study: there were no significant changes in mean pain, diarrhea, flatulence, or abdominal distension scores. In a separate study conducted by Hardman et al. (15), none of the 10 patients with dermatitis herpetiformis reported pruritus, rash, GI symptoms, or other adverse effects during a 12 weeks exposure to purified oats.

## Malabsorption

Lindsay and Moulton (7) fed four children with CD a GFD apart from the inclusion of quick porridge oat flakes. The oat flakes intake was between 46 and 169 g per day for a period of 22–96 days. Although high oat flakes intake increased fecal fat excretion, it was still within normal limits.

Tjellström et al. (16) analyzed fecal short chain fatty acids (SCFA) concentration in 116 children with newly diagnosed symptomatic CD for a 1 year period. Fifty seven of them were consuming oats containing GFD, and 59 were on a standard GFD. The SCFA patterns found in the fecal samples represent the unabsorbed fraction of SCFAs produced in the GI tract. The researchers noticed high concentrations of the pro-inflammatory acetic acid and total SCFA throughout the diet period in the GFD-oats group. This is in contrast to a significant decrease in total SCFA concentration in the GFD-standard group. No correlation was noticed between the amount of oats consumed and SCFA levels. The clinical implications of this finding are unclear. Albeit this finding, all children were in clinical and histological remission at the end of the study (except for one child in the GFD-standard group, who did not undergo a control biopsy).

As opposed to the former studies, Srinivasan et al. (17) demonstrated that lactase expression, which is an indicator of small bowel insult, was undetectable in nine patients with untreated CD, lost in patients who underwent a gluten “micro challenge,” and was normal in those on a strict GFD. Oats challenge, on the other hand, did not affect lactase activity.

## Serological Responses to Inclusion of Oats

Sey et al. (14) challenged 15 adults with CD with 350 g/week of pure, uncontaminated oats and found no serological relapses of tTG after 12 weeks of challenge. Cooper et al. (18) showed no change as well in the level of mucosal tTG and smooth muscle alpha actin expression in small bowel biopsy tissue of patients with CD after a 1 year period of oats ingestion in addition to a GFD. Furthermore, Hardman et al. (15) detected no antigliadin (IgA and IgG), antireticulin (IgA), or antiendomysial (IgA) antibodies in patients with dermatitis herpetiformis before or 12 weeks after a purified oats challenge.

Koskinen et al. (10) enrolled 23 children with CD into either an oat (45 g/day) or gluten challenge (14 g/day) in addition to oats for a period of 2 years. They showed no change in the intensity of small-bowel mucosal TG2-specific autoantibody deposits with oats ingestion. However, when wheat, rye, and barley were consumed in addition to oats, a marked small-bowel mucosal antibody response occurred in parallel with small-bowel mucosal damage within 3–12 months. When an oat-containing GFD was adopted after relapse, the small-bowel mucosal IgA deposits intensity significantly decreased within 6 months, indicating that these histological changes occurred secondary to the other cereals.

Størsrud et al. (11) reported that a large daily intake of uncontaminated rolled oats during a 2 years period did not cause any negative serological effects in adults with CD in remission. Furthermore, Janatuinen et al. (19) showed that oats did not prevent normalization of gliadin and reticulin antibodies in new onset CD nor did they cause any significant changes in gliadin antibody levels in CD patients in remission.

Picarelli et al. (20) showed that no endomysial antibodies (EMAs) were detected after 72 h of culturing CD duodenal biopsy specimens with a peptic-tryptic digest (PT) avenin. In contrast, EMAs were detected in all 13 samples after 72 h of an *in vitro* challenge with PT gliadin.

Högberg et al. (21) enrolled 116 children with newly diagnosed symptomatic CD in a randomized, double blind, multicenter study. The children were divided into two groups to receive a standard GFD with or without pure oats and were followed for a period of 12 months. The median amount of oats ingested in the GFD-oats group was 15 g daily. Ninety-two patients completed the study. There were no differences in anti-gliadin antibodies between the GFD-oats and GFD-standard groups.

## Specific Avenin Antibodies

Guttormsen et al. (22) investigated IgA antibodies against wheat gliadins, oats avenin and tTG levels in three groups of individuals: patients with CD on a GFD with (*n* = 54) or without oats (*n* = 82) and healthy individuals (*n* = 141). Both CD patient groups had equally elevated antibodies to oats compared with healthy subjects. Hvatum et al. (23) showed that patients with untreated CD have not only antibodies to wheat and oats, but also to a range of different food antigens (soy, milk proteins, and ovalbumin). Accordingly, Guttormsen et al. (22) assumed that the ongoing wheat-specific T-cell immune response affects all mucosal B cells and that the high anti-avenin antibodies in CD patients does not support a harmful effect of oats. It is worth noting that when Hvatum et al. (23) examined antibodies to different food antigens in patients with CD, they compared them to patients with various other intestinal disorders and not to a healthy population.

Hollén et al. (24) found that children with CD had developed both IgG and IgA avenin antibodies on gluten challenge. These levels positively correlated with those against gliadin and dropped to the reference level on GFD. To note, they did not challenge the patients with oats. In a later study, the same group performed serial assessments of anti-avenin antibodies in 116 children with CD on a GFD with or without 15 g (median) daily oats consumption (25). They concluded that avenin alone is not able to initiate the immune response.

## Mucosal Changes Following Oats Exposure

Janatuinen et al. (12) found no worsening of the villous architecture after 6 months of oats exposure in patients with CD in remission. In patients with newly diagnosed disease, both groups (oat consuming and GFD only) achieved similar mucosal remission. Even a longer follow-up (5 years) showed that oats ingestion does not result in any duodenal mucosal damage in adults with CD (26). In a later study they found no difference in the densities of CD3+, αβ+ intraepithelial lymphocytes (IELs), and γδ+ IELs between those consuming oats and those on a conventional GFD (27).

Koskinen et al. (10) showed that during a 2 years trial, oats had no detrimental effect on intestinal mucosal villous morphology, densities of CD3+, αβ+, and γδ+ IELs or HLADR celiac expression. Further, Högberg et al. (21) in their 1 year study mentioned above, did not find significant differences in small bowel biopsies between the GFD-oats and GFD-standard groups. The two children with an abnormal mucosa were in the GFD-standard group.

Sey et al. (14) found no significant changes in symptoms, biochemistry, or histology after oat challenge in 15 adults with CD. One patient's biopsy worsened from modified Marsh 1 to 2, likely because of non-compliance with the GFD. Another patient had a change from modified Marsh 3a to 3b.

Hardman et al. (15) examined 10 adults with dermatitis herpetiformis who consumed 50–70 g of gluten free oats daily for 12 weeks. They found no evidence of any abnormality of the villous architecture, including depth of crypts or duodenal intraepithelial lymphocytes.

In addition, in a study performed by Størsrud et al. (11) there were no changes in the villous architecture or grade of inflammation in adults with CD in remission who were taking a daily intake of 100 g of uncontaminated rolled oats for a 2 years period.

Janatuinen et al. (19) showed that oats did not prevent normalization of the number of IELs in the duodenal mucosa of newly diagnosed patients nor do they cause any significant changes in the number of IELs in CD patients in remission.

Holm et al. (28) challenged 23 children with CD in remission with either oats (median daily consumption 43–45 g/day) or gluten (median daily consumption 14 g/day). They found that after 2 years of consuming oats, all had normal densities of αβ+ IELs. In contrast, small bowel mucosal deterioration was evident within 3–12 months in all CD patients randomized to a gluten-challenge.

Kaukinen et al. (29) followed 110 adults with CD treated for up to 8 years. They found that the mucosal morphology was significantly better in subjects who had consumed larger amounts of oats or had a longer oats exposure compared to those who did not consume oats.

In contrast to the above studies, others have reported mucosal architecture damage in patients with CD consuming oats. Tuire et al. (30) followed 177 adults with CD adhering to a strict GFD for at least 2 years. They observed that persistent intraepithelial lymphocytosis was more common in long-term treated patients (42%) than in the general population (4%). At a cutoff value of 30 IEL per 100 enterocytes, oats consumption was the only factor that contributed to the persistent small-intestinal mucosal inflammation. However, at a lower cutoff value of 25 IELs per 100 enterocytes, no association between oat consumption and IEL was found.

In a study conducted by Peräaho et al. (9), the density of IEL was significantly higher in the oats consuming group, but the change in GSRS was not associated with the increase in IEL density.

Lundin et al. (31) reported a patient who showed deterioration in her small bowel histology (from Marsh type 1 to Marsh 3A lesion) during a 12 weeks challenge with 50 g of oats daily. These changes were accompanied with slight dermatitis and positive IFN-c mRNA levels. The mucosal changes resolved back to Marsh 1 after 12 weeks of a gluten and oats free diet, but recurred to Marsh 3B after a further 8 weeks oat re-challenge, accompanied with diarrhea, clinical dermatitis herpetiformis, and increased levels of IFN-c mRNA. Her mucosa failed to recover even 1 year after the re-challenge (Marsh 3A lesion). The purity of oats was tested by ELISA kit of antibody against v-gliadin, which detects barley to lesser extent. To note, this patient was one of 18 CD patients who were included in the study, whose small intestinal biopsies showed either unchanged or improved histology following oat challenge. Twelve patients who continued to consume oats beyond the challenge period had Marsh type 0–1 in their biopsies after 1.5 years of consuming oats.

## Avenin Toxicity

Real et al. (32) showed that avenins have a lower proline content compared to wheat gliadins and low molecular weight (LMW) glutenin subunits. The proline content positively correlated with the toxicity of the storage proteins of various cereals. Therefore, avenins have lower celiac toxicity with respect to wheat prolamins. In this study, however, the researchers also showed that some oat varieties could be potentially immunotoxic.

Mujico et al. (33) observed reproducible differences in the gamma-gliadin reactive T cell (which also respond to avenin peptides) stimulatory capacity of 26 oat samples. These cells are isolated from the small intestine of patients with CD and thus are strongly linked with CD. They concluded that most non-contaminated oat varieties contain avenin epitopes that are potentially harmful for a minority of the CD patient population.

Arentz-Hansen et al. (34) also demonstrated responses to TG2-treated avenin in polyclonal T-cell lines derived from the avenin-challenged biopsies from nine patients with CD, three of whom had clinical and histopathological signs of oat intolerance.

Kilmartin et al. (35) demonstrated similar immunoreactivity of CD mucosal T cell lines to protein fractions from wheat cereals, barley, rye, and oats. However, they also showed that despite their T cell line stimulation, oats do not activate a mucosal lesion in most CD patients.

## Immunogenicity of Oats

Some studies have found that oats are not immunogenic in patients with CD.

In an *in vitro* study conducted by Kilmartin et al. (36) avenin did not trigger an IFN-γ mRNA, nor did it induce significant IL-2 mRNA production in eight duodenal biopsies. This was in contrast to nine tissue samples following 4 h of culture with gliadin, where a significant increase in IFN-γ mRNA production and in IL-2 mRNA was observed.

Another *in vitro* study found that a 1 year oats challenge in patients with CD adhering to a GFD caused no change in CD8+ T cells number in the small bowel epithelium, which is a classic active CD lesion (18). In addition, oats did not alter the extent of enterocyte Ki67 expression, which reflects increased cell turnover.

Tapsas et al. (37) measured the urinary nitrite/nitrate excretion in 188 children and adolescents with CD. Increased secretion of proinflammatory cytokines and NO metabolites precedes the mucosal alterations, thus it is considered a reliable indicator of small intestinal inflammation. There was no correlation between urinary NO metabolites and oats consumption or duration in the GFD.

Srinivasan et al. (38) examined expression of immunological molecules, considered to reflect immune activation in small bowel tissue, of 10 CD patients taking 50 g of oats daily for 12 weeks. They found no increase in major histocompatibility complex (MHC) class II molecule, CD25 positive cells, the intercellular adhesion molecule 1 (ICAM-1), the nuclear proliferating antigen Ki-67 or mast cell enzyme tryptase expression. Moreover, MHC class II staining developed and numbers of CD25 positive cells increased when the patients were given a gluten challenge.

Hardy et al. (39) assessed whether oats ingestion or other known toxic grains in CD stimulate an avenin-specific T cell response *in vivo*. They fed participants oatmeal (100 g/day over 3 days) to measure the *in vivo* polyclonal avenin-specific T cell responses to peptides contained within comprehensive avenin peptide libraries in 73 HLA-DQ2.5(+) patients with CD. Grain cross-reactivity was investigated using an oral challenge with wheat, barley, and rye. Avenin-specific responses against four closely related peptides were observed in six (8%) of the 73 subjects.

Sjöberg et al. (40) studied the same group of patients that Högberg et al. (21) had enrolled and analyzed changes in mRNAs expression levels of for immune effector molecules and tight junction proteins in small intestinal biopsies from patients treated with GFD+oats and standard GFD, respectively, before and after the intervention. The researchers found that mRNA levels for all five chemokines tested (CX3CL1, CXCL8/IL-8, CXCL9, CXCL10, and CXCL11), followed disease activity with no difference between the two GFD diets. On the other hand, when they tested proinflammatory and downregulatory cytokines, they found that mRNA for IL-10, TGF-β1, and TNF-α, did not normalize in several patients on oats-containing GFD. Regarding NK receptors and MHC class I molecules, KLRC2/NKG2C and KLRC3/NKG2E mRNA levels normalization did not occur to the same extent on the GFD+oats diet as on the GFD-std diet. Some individuals even showed increased levels. As far as for tight junction proteins, CLDN-4 mRNA levels were higher in active CD than in controls and showed significant decline after GFD-std but not after GFD+oats. The researchers concluded that there was ongoing immune activity in the intestinal mucosa of patients in the GFD+oats group. More studies implied the presence of immunogenicity in some varieties of avenins.

The mucosal level of IFN-c mRNA in treated CD patients on a standard gluten and oats free diet is usually below 1 × 10^3^ transcripts per μg total RNA. Lundin et al. (31) found that after an oats provocation period, five of 19 patients with CD had detectable levels of IFN-c mRNA. One of these five also had a positive level before the provocation, and one patient was tested only afterwards.

Silano et al. (3) demonstrated that avenins were able to activate peripheral lymphocytes from patients with CD, although avenins from different cultivars displayed different immunogenic activity. In another study, this group indicated that some varieties of oats may be potentially harmful to individuals with CD (41). The differences may be partially explained by the fact that oat immunogenicity depends on the cultivar used.

Comino et al. (42) showed that there are differences in immunogenicity of various oat varieties, and that some of them are not reactive against the T cells of patients with CD. They reported the G12 antibody as competent to identify potentially toxic oat varieties for patients with CD. In another study, though, this group found the existence of new potentially toxic peptides. These peptides were able to activate circulating dendritic cells (the most potent antigen-presenting cells of the immune system that have the capacity to trigger T-cell proliferative responses from patients with CD) (43).

## Quality of Life

In a 2 years intervention study conducted by Størsrud et al. (44), 15 adults with CD consumed an average of 93 g of rolled oats daily. All patients commented on the beneficial effects of oats, i.e., a better taste, the satiating effect, more variability and improved bowel function. These effects were independent of the amount of oats consumed.

Peraaho et al. (9) found that oats had no effect on quality of life (QOL) as measured by the Psychological General Well-Being (PGWB) questionnaire. In another study, this group surveyed 494 patients with CD who were on an oat-containing GFD: 94% of them felt that oats diversified their GFD and was in many respects beneficial (45).

Aaltonen et al. (46) followed a large cohort of 869 patients with CD for a 1 year period. They demonstrated that patients with CD consuming oats(all aged over 16 years) as part of a longstanding GFD had similar or even somewhat better QOL than those not consuming oats. Further, there was no difference between the groups in symptoms, CD serology, and small-bowel mucosal damage after 1 year on a GFD.

## Wheat Contamination of Oat Products

The fact that oats are often processed on the same production line as wheat raises the possibility of gluten contamination. Gimenez et al. (47) examined 132 oat accessions and showed that 73% of them could be considered “gluten-free” (in the range 3–20 ppm). Mujico et al. (33) in contrast to the previous group observed that commercially ground oat flours were mostly contaminated with other cereals.

A study conducted in Spain analyzed 108 oat samples (e.g., rolled oats, oat flakes, and flours) collected from Europe, the United States, and Canada (48). Three quarters of the samples were contaminated with more than 20 ppm of gluten: with variation of up to 8,000 ppm.

In the USA, 9 out of 12 containers of rolled or steel-cut oats, representing four different lots of each of three separate brands (Quaker, Country Choice, and McCann's) had gluten levels from 23 to 1,807 ppm. All three brands of oats had gluten levels above 20 ppm (49).

## How Much Oats is Good Enough?

The tolerance threshold for gliadin has been evaluated to be 20 ppm (50–53). The daily intake of oats is considered to be 40–80 g, corresponding to 0.2–1.2 g avenin, which is about 10% of the gliadin toxicity amount. On that basis it can be speculated that a much higher amount (about 10 times) than the average daily intake of oats is required to induce a toxic mucosal reaction (2).

A Health Canada publication recommended that the amount of uncontaminated oats consumed by individuals with CD should be limited to 20–25 g/day for children and 50–70 g/day for adults (1).

## What Kind of Oats?

A study conducted by Kemppainen et al. (54), in which 33 patients with CD consumed more than 90 g daily of either kilned or unkilned oats for 12 months, demonstrated that adding high amount of kilned oats into the GFD led to increased vitamin B1 intake.

According to the American College of Gastroenterology (ACG) clinical guidelines, oats should be introduced into the diet with caution and patients should be monitored closely for evidence of any adverse reaction (strong recommendation) (55). In addition, according to the ESPGHAN clinical guideline, unless the purity of the oats can be guaranteed, their safety remains questionable (56).

## Summary

A large number of reviews have been published on the matter of the safety of oats in GFD. Most of these reviews concluded that pure oats are well-tolerated by most CD patients (4, 55, 57–61), at moderate amounts (20–25 g/day dry rolled oats for children; 50–70 g/day for adults) (1, 62) or even up to 100 g/day (63). Inclusion of oats in a GFD might be valuable due to their nutritional and health benefits and improvement of food variety (64, 65). Nevertheless, since the potential for sensitivity/toxicity exists (2, 58), oats should be added with caution to a GFD (55, 57), only after all CD symptoms including weight loss and growth disturbances have resolved (1), after at least 6 months of a conventional GFD (1, 4, 66) and, in our opinion, also only after normalization of serology. The need for pre-exposure biopsy is unclear and should be considered on an individual basis. These patients should be closely monitored for evidence of intolerance (1, 4, 55, 66) and some even suggest annual screening biopsies to ensure that subclinical changes are not occurring in the small bowel mucosa (57). Oats contamination by gluten must always be considered (4, 56, 63, 67), mandating a continuous need to ensure oats purity (56, 60, 67, 68).

## Author Contributions

IS reviewed the literature and wrote the manuscript. RS reviewed the literature and helped with the writing of the manuscript. AD reviewed the manuscript and helped with the writing of the manuscript.

### Conflict of Interest

The authors declare that the research was conducted in the absence of any commercial or financial relationships that could be construed as a potential conflict of interest.
